# Supplementary material for: Preparation of Boron Nitride Nanoplatelets via Amino Acid Assisted Ball Milling: Towards Thermal Conductivity Application
Source: Nanomaterials (Basel). 2020 Aug 22;10(9):1652. doi: 10.3390/nano10091652 (PMC7557982; doi:10.3390/nano10091652)
Supplement: Supplementary file 1 [file nanomaterials-10-01652-s001.pdf]

nanomaterials

Supporting information

Preparation of Boron Nitride Nanoplatelets via Amino Acid Assisted Ball Milling: Towards Thermal Conductivity Application

Nan Yang [1,†], Haifeng Ji [1,†], Xiaoxia Jiang [2], Xiongwei Qu [1], Xiaojie Zhang [1,*], Yue Zhang [1,*] and Binyuan Liu [1,*]

1. Hebei Key Laboratory of Functional Polymers, Department of Polymer Materials and Engineering, Hebei University of Technology, 8 Guangrong Street, Tianjin 300130, China; 201721504016@stu.hebut.edu.cn (N.Y.); 201911501019@stu.hebut.edu.cn (H.J.); xwqu@hebut.edu.cn (X.Q.)
2. School of Chemical Engineering and Technology, Hebei University of Technology, 8 Guangrong Street, Tianjin 300130, China; jiangxx@hebut.edu.cn

* Correspondence: zhangxj@hebut.edu.cn (X.Z.); zylizzy@hebut.edu.cn (Y.Z.); byliu@hebut.edu.cn (B.L.); Tel.: +86-13843143643 (X.Z.)

† These authors contributed equally.

1. X-ray photoelectron spectroscopy (XPS) measurements

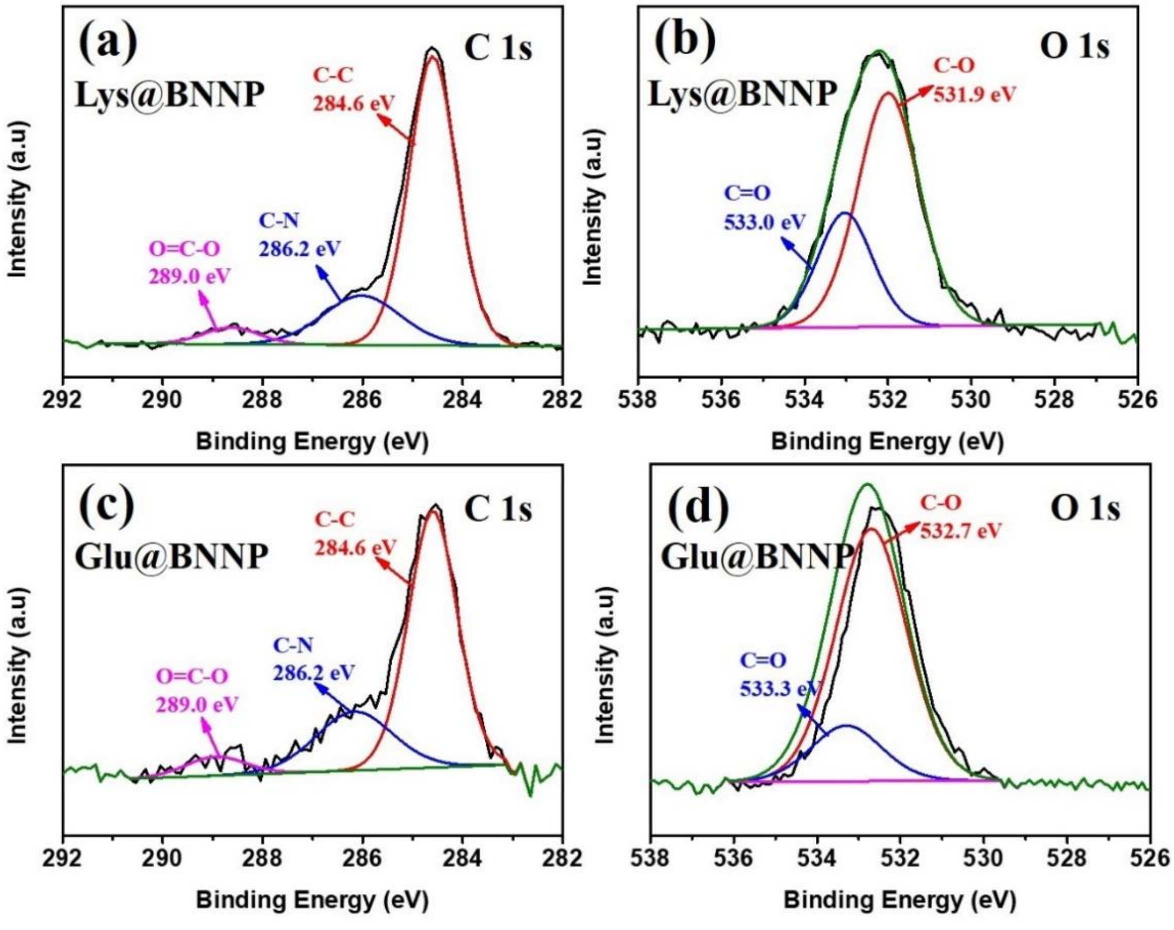

Figure S1. (a)-(b) and (c)-(d) C 1s and O 1s core-level spectra of Lys@BNNP and Glu@BNNP.

2. Transmission electron microscopy

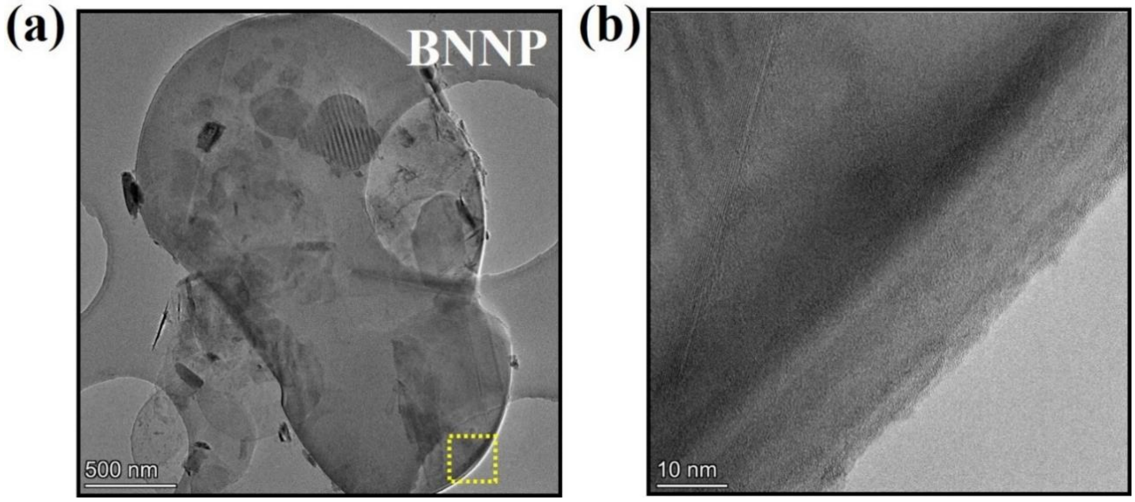

Figure S2. (a) Low-magnification TEM image of BNNP (b) High-resolution TEM images of the BNNP.

3. Raman spectroscopy

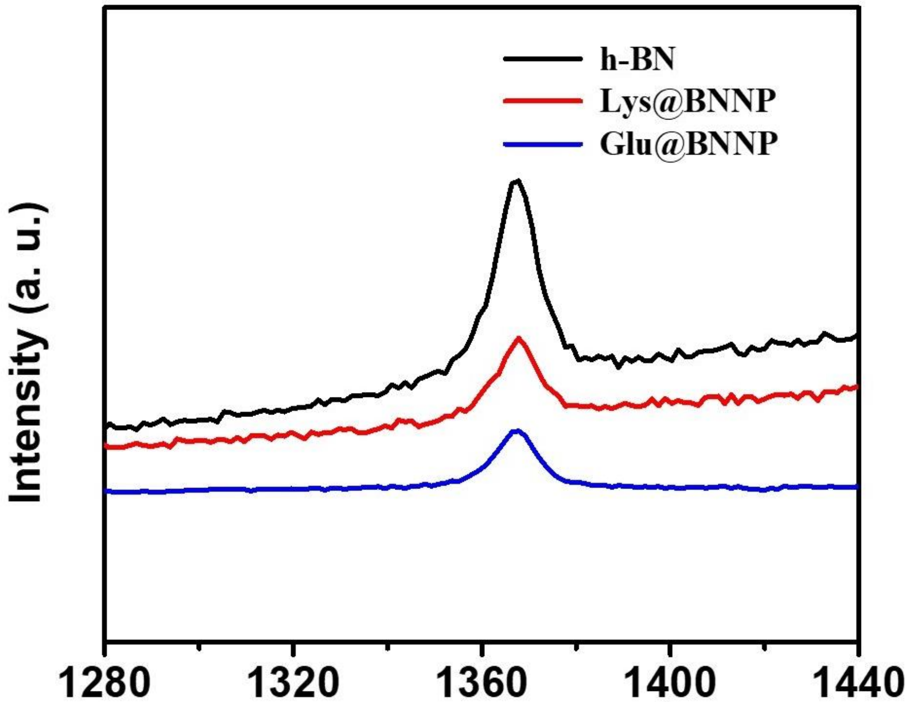

Figure S3. Raman spectroscopy of as-received h-BN, Lys@BNNP and Glu@BNNP.

4. Mechanical properties of BNNP/PVA hydrogels

PVA hydrogel

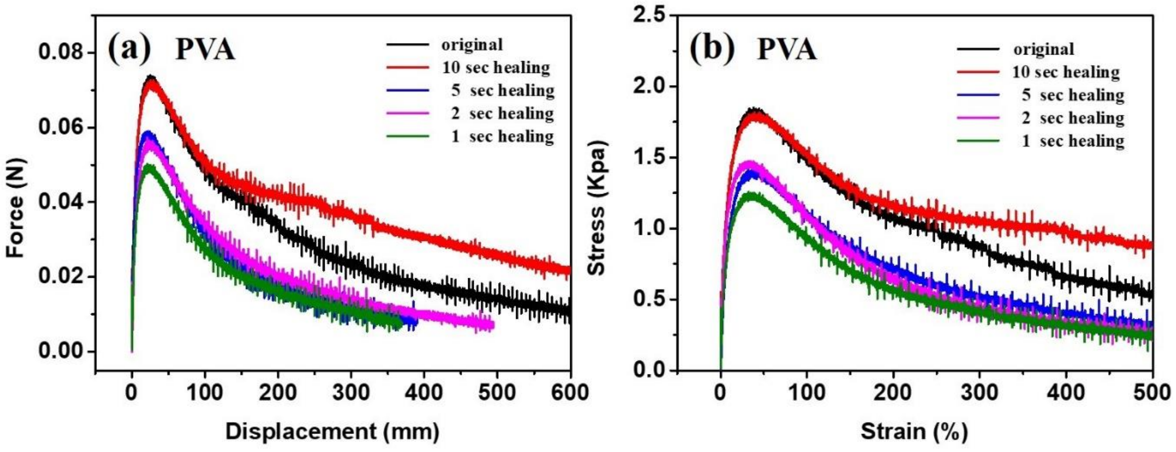

Figure S4. (a) Force-displacement curve of original PVA hydrogel and the healed PVA hydrogels, (b) Tensile stress-strain curve of original PVA hydrogel and the healed PVA hydrogels.

BNNP/PVA hydrogel

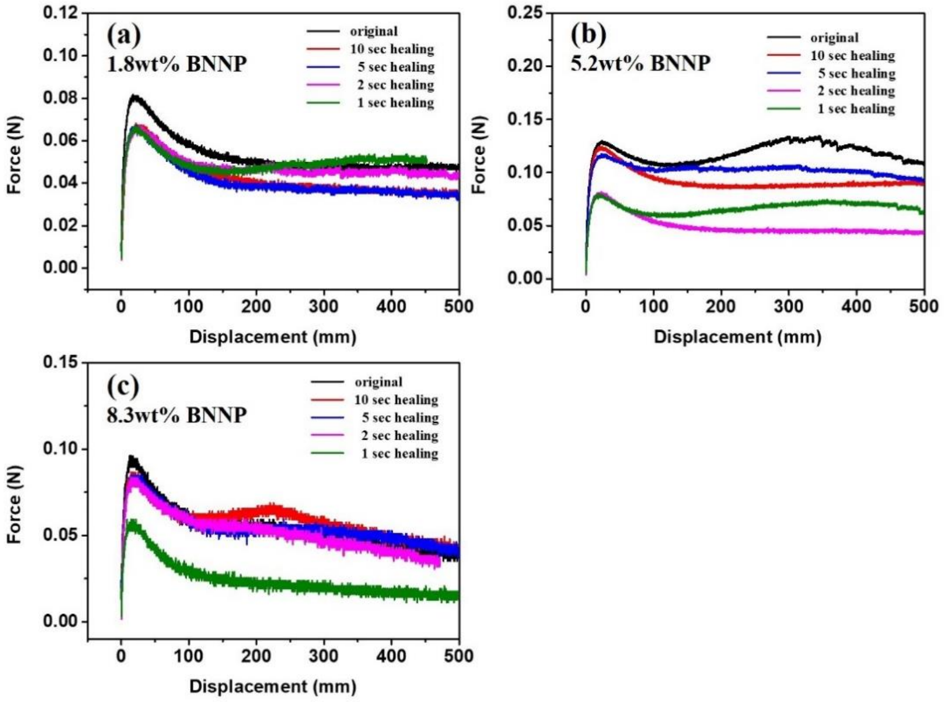

Figure S5. Force-displacement curve of BNNP/PVA hydrogels with different contents of BNNP (1.8, 5.2 and 8.3 wt%), and the healed BNNP/PVA hydrogels (a) 1.8 wt%, (b) 5.2 wt%, (c) 8.3 wt%.

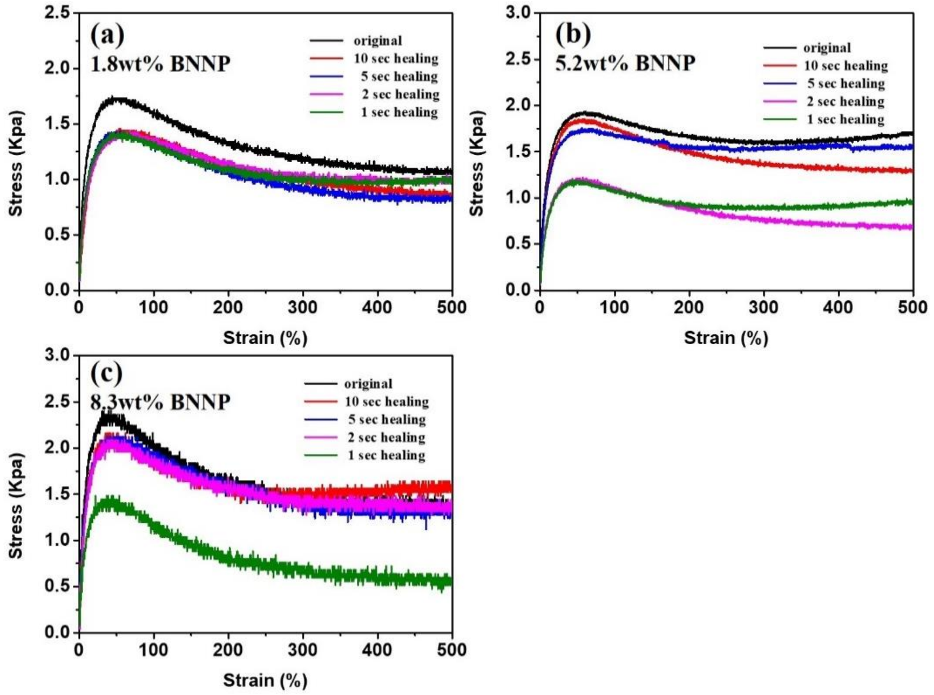

Figure S6. Tensile stress-strain curve of BNNP/PVA hydrogels with different contents of BNNP (1.8, 5.2 and 8.3 wt%), and the healed BNNP/PVA hydrogels (a) 1.8 wt%, (b) 5.2 wt%, (c) 8.3 wt%.

Lys@BNNP/PVA hydrogel

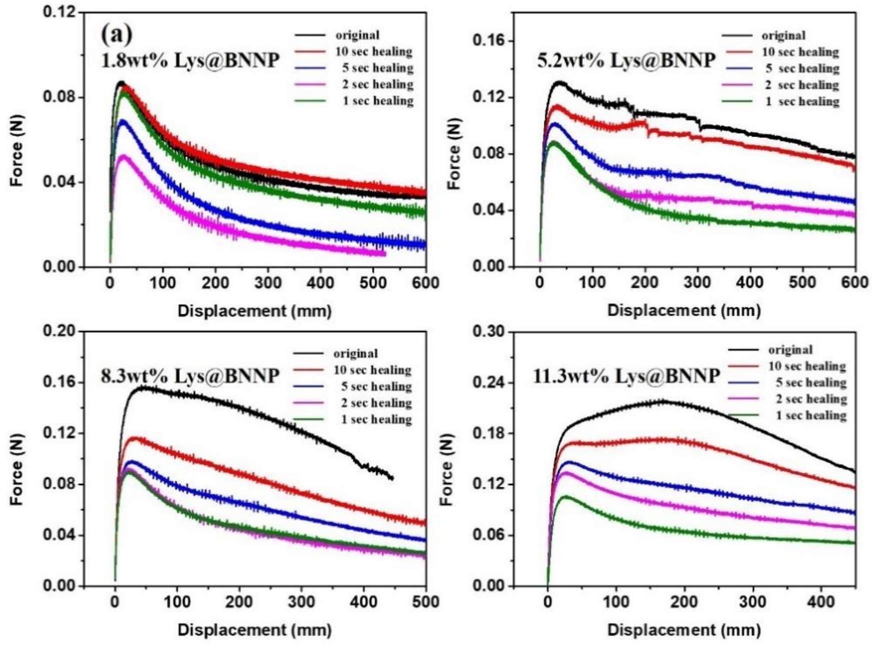

Figure S7. Force-displacement curve of Lys@BNNP/PVA hydrogels with different contents of Lys@BNNP (1.8, 5.2, 8.3 and 11.3 wt%), and the healed Lys@BNNP/PVA hydrogels (a) 1.8 wt%, (b) 5.2 wt%, (c) 8.3 wt% and (d) 11.3 wt%.

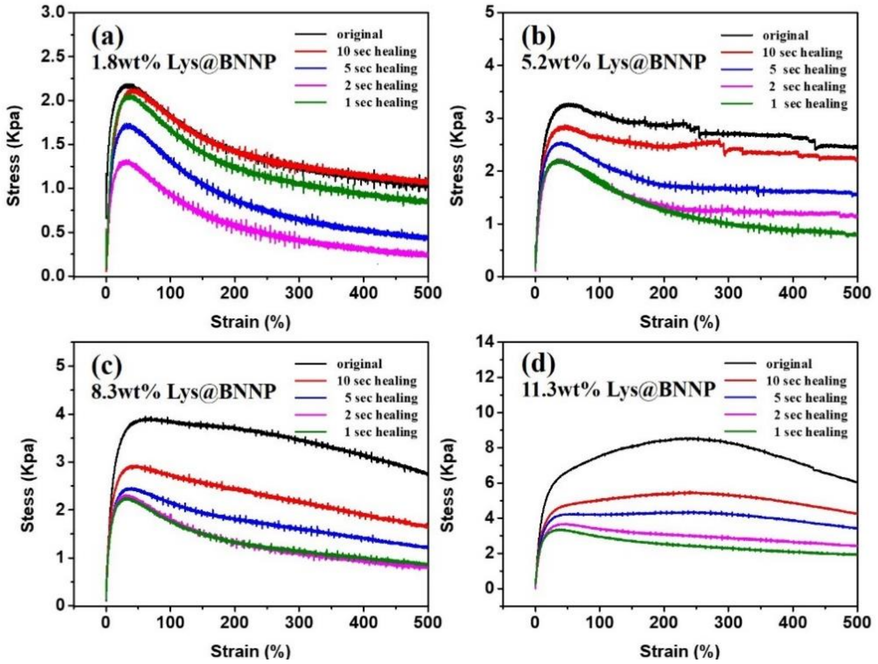

Figure S8. Tensile stress-strain curve of Lys@BNNP/PVA hydrogel with different contents of Lys@BNNP (1.8, 5.2, 8.3 and 11.3 wt%), and the curves of healed Lys@BNNP/PVA hydrogels (a) 1.8 wt%, (b) 5.2 wt %, (c) 8.3 wt% and (d) 11.3 wt%.

Glu@BNNP/PVA hydrogel

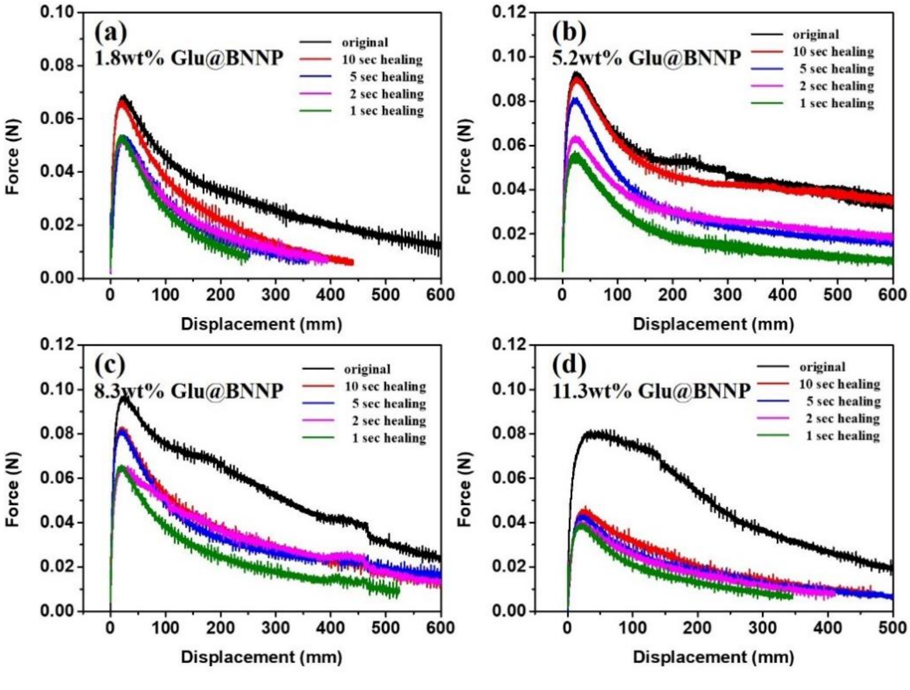

Figure S9. Force-displacement curve of original Glu@BNNP/PVA hydrogel with different contents of Glu@BNNP (1.8, 5.2, 8.3 and 11.3 wt%), and the curves of healed Glu@BNNP/PVA hydrogels (a) 1.8 wt%, (b) 5.2 wt%, (c) 8.3 wt% and (d) 11.3 wt%.

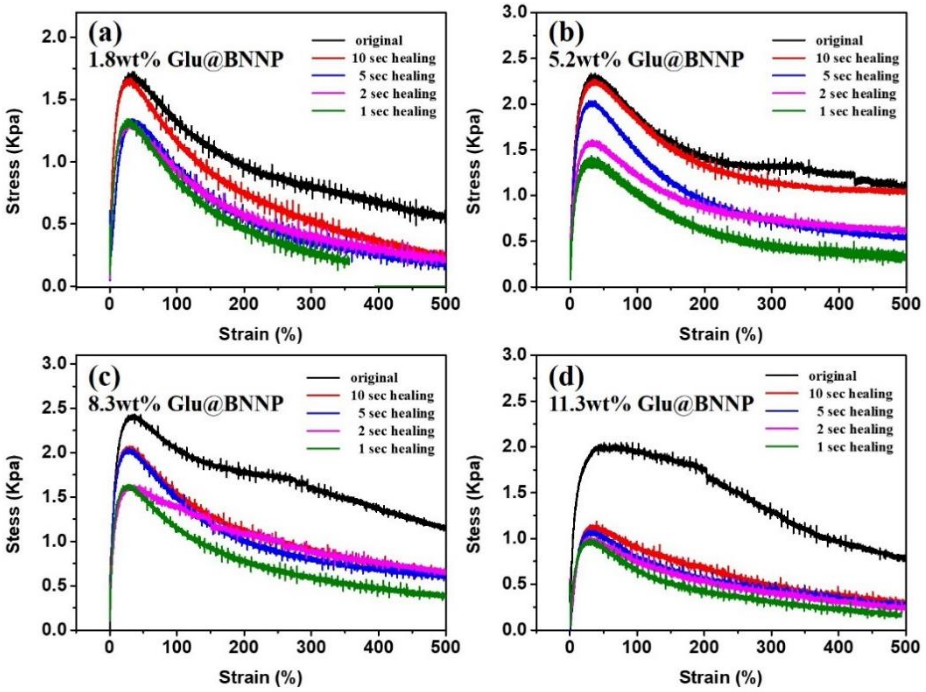

Figure S10. Tensile stress-strain curve of original Glu@BNNP/PVA hydrogel with different contents of Glu@BNNP (1.8, 5.2, 8.3 and 11.3 wt%), and the curves of healed Glu@BNNP/PVA hydrogels (a) 1.8 wt%, (b) 5.2 wt%, (c) 8.3 wt% and (d) 11.3 wt%.

The self-healing test process

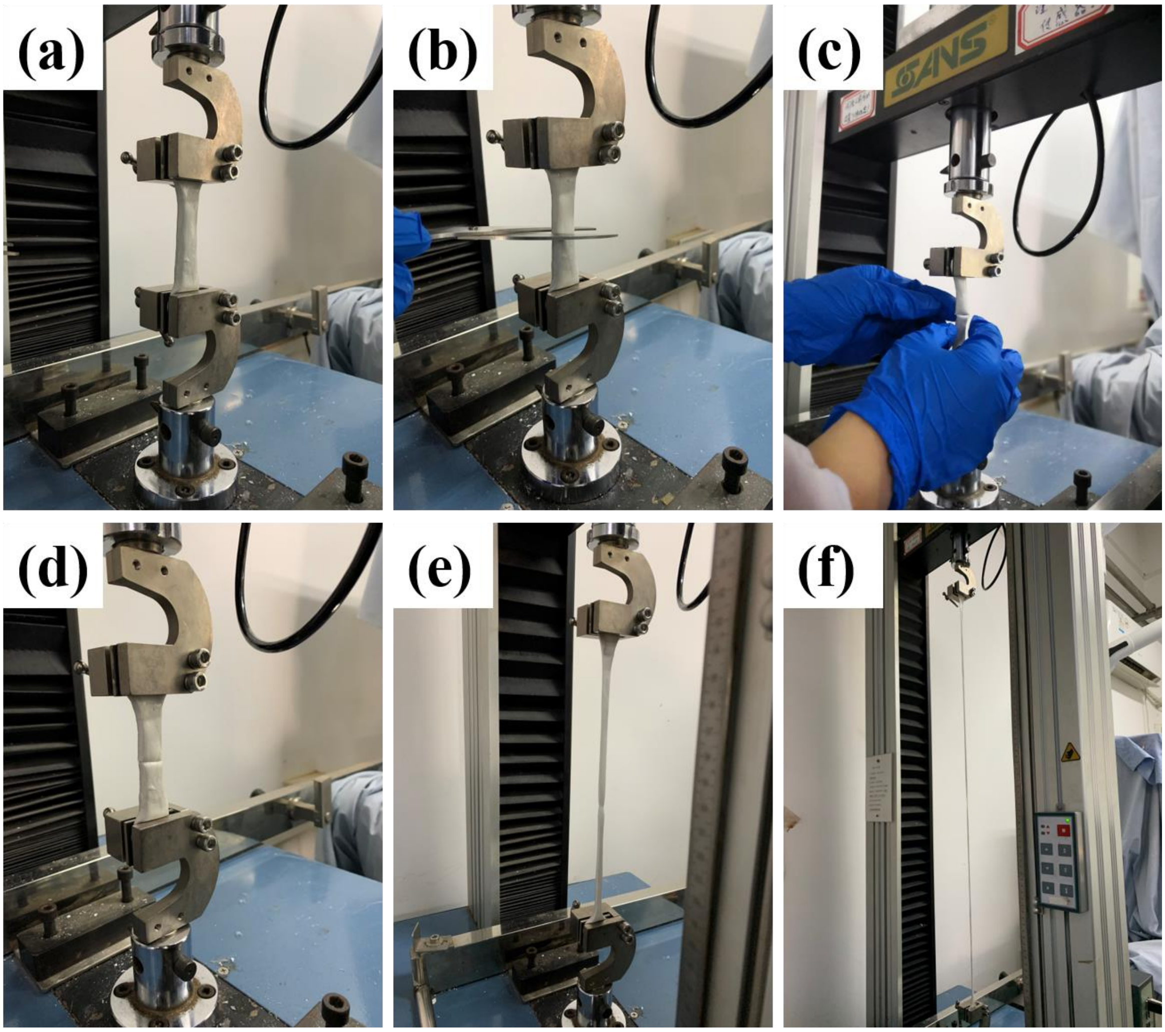

Figure S11. The self-healing test process of Strip-shaped hydrogel samples. In order to test the rapid self-healing performance of the BN samples, self-healing tests were carried out directly on the electronic universal testing machine. (a) Strip-shaped sample was clamped on the testing machine; (b) the hydrogel is cut into two pieces; (c) the two pieces of the hydrogel are re-contacted; (d) the hydrogel can self-heal automatically after contacting for 1 s, 2 s, 5 s or 10 s at room temperature; (e) the healed hydrogel is being tensile tested; and (f) end of healed hydrogel tensile test.
